# Cefepime-induced neurotoxicity

**DOI:** 10.4102/sajid.v40i1.704

**Published:** 2025-04-30

**Authors:** Jessica Taylor, Hannah M. Gunter, Karen Cohen

**Affiliations:** 1 Division of Clinical Pharmacology, Department of Medicine, Faculty of Health Sciences, University of Cape Town, Cape Town, South Africa

**Keywords:** cefepime, neurotoxicity, cefepime-induced neurotoxicity, adverse drug reaction, encephalopathy

## Abstract

We present a case of severe cefepime-induced neurotoxicity following acute kidney injury in a patient with a fracture-related infection. Despite stopping cefepime, the patient required intubation, ventilation, multiple antiepileptic drugs for seizure control, and haemodialysis.

**Contribution:**

Cefepime-induced neurotoxicity is a reversible cause of encephalopathy. Early recognition and cefepime withdrawal are crucial. As cefepime use in South Africa increases due to antimicrobial resistance, healthcare workers must be aware of this under-recognised, under-reported serious adverse drug reaction.

## Introduction

Cefepime, a fourth-generation cephalosporin, has a broad spectrum of activity against Gram-positive and Gram-negative bacteria, including *Pseudomonas* spp.^1,2^ Cefepime exerts its bactericidal activity via inhibition of bacterial cell wall synthesis.^1^ The drug’s resistance to hydrolysis by several of the beta-lactamases makes it an important carbapenem-sparing agent. Reports of cefepime-induced neurotoxicity date back to 1999.^3^ In prospective cohort studies that excluded critically ill patients and those with pre-existing neurological disorders, neurotoxicity occurred in 1% – 2.5% of the patients treated with cefepime.^4,5^ In a cohort of critically ill patients treated with cefepime, many of whom were elderly, 15% developed cefepime-induced neurotoxicity.^6^ Recognition of this adverse drug reaction (ADR) is poor and often delayed because of several competing causes of encephalopathy in this patient population.^3^ Cefepime-induced neurotoxicity is a reversible cause of encephalopathy. Early recognition and cessation of cefepime are key to successful management. We present the following case to increase healthcare worker awareness of this under-reported and preventable ADR.

## Case presentation and management

A 38-year-old woman with HIV and in month 6 of drug-sensitive tuberculosis (TB) treatment on rifampicin and isoniazid was admitted to hospital with extensive injuries following a pedestrian vehicle accident. Her antiretroviral therapy was interrupted on admission to the intensive care unit, as she was unconscious, and her HIV status was not reported in the collateral history. She sustained a tibia–fibula fracture which was complicated by a fracture-related infection caused by *Enterobacter cloacae*, sensitive to cefepime and gentamicin. Directed therapy with 2 g of intravenous cefepime 8-hourly was initiated, with an intended treatment duration of 6 weeks. She had normal renal function at baseline (Table 1).

**TABLE 1:** Renal function results and timeline of clinical events.

Test [Reference range]	Admission to hospital	Cefepime started	Onset of nausea and vomiting	Onset of symptoms of neurotoxicity	Seizures	Cefepime stopped on day 37	Admitted to ICU Haemodialysis started	Extubated	Discharged
			Day 30 of cefepime	Day 34 of cefepime	Day 37 of cefepime		Day 3 after cefepime stopped	Day 18 after cefepime stopped	Day 37 after cefepime stopped
Sodium (mmol/L) [136–145]	135	133	136	138	146	-	142	134	140
Potassium (mmol/L) [3.5–5.1]	3.2	4.1	2.5	3.3	3.2	-	3.7	5.6	3.5
Urea (mmol/L) [2.1–7.1]	3.1	3.3	8.1	14.6	17.9	-	20.1	11.8	3.9
Creatinine (μmol/L) [49–90]	52	40	73	177	293	-	506	179	86
eGFR (CKD-EPI) (mL/min/1.73 m^2^)	116	127	90	31	17	-	9	30	74

ICU, intensive care unit; eGFR, *estimate glomerular filtrate rate;* CKD-EPI, chronic kidney disease epidemiology.

On day 30 of cefepime therapy, she developed nausea and vomiting, followed by acute deterioration in renal function (Table 1). She became withdrawn, progressing to aphasia and refusing oral intake. On day 34 of cefepime, she developed generalised tonic-clonic seizures and facial muscle myoclonus involving the periocular muscles. Treatment with multiple antiepileptic drugs, including benzodiazepines, phenytoin, levetiracetam, carbamazepine, and sodium valproate, was initiated. Cefepime was stopped on day 37, as cefepime-induced neurotoxicity was suspected. Isoniazid and rifampicin were also stopped, as she had completed 6 months of TB treatment. The following differential diagnoses for encephalopathy were considered and excluded: uraemia, sepsis, meningitis, encephalitis, isoniazid-induced neurotoxicity, HIV-related opportunistic infection, and other intracranial pathology. Relevant results of the diagnostic workup are shown in Table 2. Further TB work-up, including urine lipoarabinomannan (U-LAM), sputum TB GeneXpert® and culture, found no evidence for TB. Computed tomography of the brain was normal. Therapeutic drug monitoring (TDM) of cefepime is not available at the treating facility.

**TABLE 2:** Relevant laboratory results.

Specimens	Tests	Results [Reference range]
Cerebrospinal fluid	Appearance and clarity	Clear, no bacteria observed, supernatant colourless
	Glucose	3.6 mmol/L
	Protein	1.72 g/L [0.15 g/L – 0.45 g/L]
	Cell count	No cells detected
	Polymorphonuclear cells	Lower than detectable
	Lymphocytes	Lower than detectable
	Red cells	Lower than detectable
	Cryptococcal antigen test	Negative
	Bacterial culture	No growth after 3 days
	Mycobacterial culture liquid medium	No growth after 42 days
	FTA-ABS (treponemal antibodies)	Negative
Blood	CRP	18 mg/L [<10 mg/L]
	CD4 count	252 cells/µL
	Bacterial culture	No growth after 5 days
Urine	Microscopy	Scanty leucocytes, scanty erythrocytes, and yeast cells observed
	Bacterial culture	<10 000 cfu/mL isolated

FTA-ABS, fluorescent treponemal antibody absorption test; CRP, c-reactive protein; cfu, colony-forming unit.

The patient’s renal function and level of consciousness deteriorated despite discontinuing cefepime. On day 2 after stopping cefepime, she was intubated and ventilated for hypoxic respiratory distress secondary to aspiration pneumonia. Intravenous meropenem, at a dose of 2 g 8 hourly, was initiated. Sustained low-efficiency dialysis was commenced to enhance elimination of cefepime. Neurological improvement occurred 9 days after stopping cefepime and 6 days after initiation of dialysis. Her admission was further complicated by a urinary tract infection with *Enterococcus faecalis* that required treatment with intravenous ampicillin. She was extubated on day 18, and dialysis was stopped on day 19 after stopping cefepime. Renal biopsy was not performed. She was discharged to a step-down facility 3 weeks later, after reinitiating antiretroviral therapy. Antiepileptic drugs had been stopped with no further seizures noted. Her creatinine was on a downward trend (Table 2). On discharge, she was fully orientated and was mobilising with a walking aid.

## Discussion

Beta-lactam antimicrobials such as penicillins, cephalosporins, and carbapenems have a wide therapeutic interval and are generally considered safe. However, all have been associated with neurotoxicity.^7^ This propensity to induce neurotoxicity is because of the structural similarity between the neurotransmitter gamma-aminobutyric acid (GABA) and the beta-lactam ring.^8^ Binding of endogenous GABA to the GABA_A_ receptor complex leads to an intracellular influx of chloride ions that hyperpolarises the neuron, creating an inhibitory post-synaptic potential.^7^ Because of their structural similarity to GABA, beta-lactam antimicrobials act as antagonists at the GABA_A_ receptor complex, preventing binding of endogenous GABA and subsequent ion conduction in a concentration-dependent manner.^7,9^ Beta-lactam antimicrobials also decrease endogenous GABA release and increase excitatory neurotransmitter release from pre-synaptic nerve terminals and inhibit the activity of benzodiazepine receptors.^7^ The net effect is central hyperexcitation, causing myoclonus, encephalopathy, and seizures.^7^ Although all beta-lactams have neurotoxic potential, the risk is highest with cefepime and cefazolin.^7,10^

Cefepime-induced neurotoxicity is caused by excessive central nervous system (CNS) concentrations of the drug, which results from increased plasma concentrations because of excessive dosing and/or reduced renal clearance. Cefepime plasma trough concentrations >20 mg/L have been associated with a fivefold increased risk of cefepime-induced neurotoxicity.^11^ Cefepime relies mainly on renal elimination, and dose adjustment is required in renal impairment (Table 3).^1^

**TABLE 3:** Cefepime dosage in renal failure.

Usual dosages	Recommended maintenance dosages		
CrCl > 60 mL/min*	CrCl 30 mL/min – 60 mL/min	CrCl 11 mL/min – 29 mL/min	CrCl <11 mL/min
2 g 8 hourly	2 g 12 hourly	2 g 24 hourly	1 g 24 hourly
2 g 12 hourly	2 g 24 hourly	1 g 24 hourly	500 mg 24 hourly
1 g 12 hourly	1 g 24 hourly	500 mg 24 hourly	250 mg 24 hourly
500 mg 12 hourly	500 mg 24 hourly	500 mg 24 hourly	250 mg 24 hourly

*Source*: Adapted from CEFIPIME FOR INJECTION [package insert]. Lake Forest, IL: Hospira Inc.; 1996 (revised 2019). [cited 2025 Mar 11]. Available from: https://labeling.pfizer.com/ShowLabeling.aspx?id=13898

CrCl, creatinine clearance.

* , Creatinine clearance (mL/min) estimated using the Cockcroft–Gault equation.

Consequently, renal impairment is associated with an increased risk of cefepime-induced neurotoxicity. In a systematic review of 135 case reports, 80% of the cases of cefepime-induced neurotoxicity occurred in patients with underlying renal dysfunction, and 48% of the cases had received excessive doses for their reported renal function.^3^ Renal dysfunction may cause increased blood–brain barrier permeability of cefepime because of alterations in protein binding and the accumulation of organic acids that compete with efflux transporters in the CNS.^7^ As seen in our patient, cefepime-induced neurotoxicity has most commonly been described where inappropriately large doses of the drug are administered to patients with renal dysfunction. However, up to 20% of the cases of cefepime-induced neurotoxicity may have no underlying renal dysfunction, and 26% of the cases develop toxicity despite appropriate doses for renal function.^3^ In these cases, increased CNS cefepime concentrations result from increased CNS penetration from systemic inflammation and blood–brain barrier dysfunction. Additional risk factors for cefepime-induced neurotoxicity include advanced age, pre-existing neurological disease, and, specific to our patient, prolonged treatment duration.^2,9,12^ We did not determine the specific cause of renal dysfunction in our patient, and her renal function improved before a renal biopsy could be performed. Dehydration from nausea, vomiting, and poor oral intake may have contributed to the development of acute tubular necrosis. Acute interstitial nephritis (AIN), a known class effect of cephalosporins, is another consideration, but reports of cefepime-induced AIN are rare.^1,13,14,15^ Our patient was taking standard doses of isoniazid, a drug with neurotoxic potential. Acute overdoses of isoniazid may present with seizures and encephalopathy, but these are unlikely during chronic therapy at conventional doses.^16^ There are case reports describing encephalopathy in patients on isoniazid with end-stage kidney disease receiving regular haemodialysis. In these cases, encephalopathy is thought to be related to haemodialysis-induced severe deficiency of pyridoxal phosphate (the active form of pyridoxine), rather than reduced renal clearance.^17,18^ Therefore, we think that isoniazid is unlikely to have been implicated in our patient’s encephalopathy.

Cefepime-related neurotoxicity presents with an altered mental state, disorientation or agitation that progresses to aphasia, myoclonus, coma, and occasionally seizures.^3,6,19^ Initial signs and symptoms are often subtle and difficult to detect in the critically ill patient. The diagnosis requires a high index of suspicion and exclusion of alternative causes. Myoclonus is a common presentation, often involving the facial and periocular muscles, similar to our case.^6^ Seizures are not usually the primary manifestation of neurotoxicity, and few patients will present with isolated seizures.^6,19^ Beta-lactam neurotoxicity may also present with or be complicated by non-convulsive status epilepticus.^2,20^ A typical electroencephalogram (EEG) pattern of diffuse background slowing with or without generalised periodic discharges with triphasic morphology has been described in patients with cefepime-induced neurotoxicity.^6,21^ This EEG pattern is not specific to cefepime and may be present in other toxic encephalopathies.^21^ While elevated plasma concentrations of cefepime strongly support the diagnosis of cefepime-induced neurotoxicity, the laboratory assay to quantify cefepime concentrations is not available in the South African public healthcare sector.

The primary management of cefepime-induced neurotoxicity is discontinuation of the drug. Where ongoing antimicrobial therapy is required, avoiding beta-lactams where possible or using beta-lactams with the lowest neurotoxic potential is advised.^10^ Based on the mechanism of neurotoxicity, allosteric GABA agonists such as the benzodiazepines and barbiturates are considered first-line in the management of seizures.^9,22^ There are no comparative studies to inform this recommendation, and various other antiepileptic drugs have been used in published case reports.^3^ In patients with normal or recovering renal function, stopping cefepime, controlling seizures, and supportive care may be sufficient for recovery.^3^ In most cases, neurotoxicity will improve within 5 days of drug withdrawal.^2^ Ongoing recovery has been reported up to day 10, such as in our patient, and even later in isolated cases.^2,4,23^ Haemodialysis may be indicated in patients with severe renal impairment or refractory neurotoxicity.^3,20^ The pharmacokinetic properties of cefepime, including its low molecular weight, low volume of distribution, and limited protein binding, make it amenable to removal by haemodialysis.^24^ A single 3-h haemodialysis session may efficiently reduce serum cefepime concentrations by 70%.^24^ Case reports suggest that time to recovery may be faster following withdrawal of cefepime and initiation of haemodialysis (from 12 h to 7 days) compared to withdrawal of cefepime alone (1–17 days).^24^ Although often overlooked, it is essential to report the ADR once recognised. Clinicians are encouraged to report all suspected ADRs, particularly those that are serious (i.e. causing or prolonging hospitalisation or resulting in mortality), even when they have been previously described. This allows for enhanced drug safety awareness among healthcare workers and an accurate assessment of the risk–benefit ratio of drugs.

## Conclusion

Cefepime-induced neurotoxicity is a reversible cause of encephalopathy. An ADR should be considered in the differential diagnosis of any patient who develops an altered mental state after receiving treatment with a beta-lactam antimicrobial. Identifying patients at risk of cefepime-induced neurotoxicity, such as those with renal impairment, and applying appropriate dose adjustments may prevent the development of cefepime-induced neurotoxicity. Regular monitoring of renal function and performance of serial neurological examinations are recommended in patients treated with cefepime, particularly those with risk factors for neurotoxicity, so that neurotoxicity is recognised early and cefepime stopped.
